# Professional Fees

**Published:** 1864-10

**Authors:** L. C. Ingersoll

**Affiliations:** Keokuk, Iowa


### PROFESSIONAL FEES.

**An Essay read before the Iowa State Dental Society at Davenport, Iowa, August 10, 1864.**

BY L. C. INGERSOLL.

In presenting an essay on this subject it is not my purpose to offer a *fee bill*—but to state some general principles, and to show the relation of Dental Fees to Dentistry.

Money can never be made a measure of values. The value of bread, water, apparel, education, nor any thing necessary in securing these, can be counted in dollars and dimes. A dime may purchase that which will be of a value to me beyond any computation. In a moment of time of trifling importance, it is possible to render incalculable service to a fellow mortal. Hence it will be seen that in the price paid for any thing there is no aim at rendering an equivalent—that no graduation of prices either in commercial transactions or professional services, can ever be made on the scale of equivalents. Prices, therefore, are merely nominal in respect of value. Value is the estimate which the community put upon one thing proportionately with other things. And the price paid is an expression, relatively, of that estimate. One places a high estimate on the teeth, and expresses that estimate by a willingness to pay two, three, five, or twenty-five dollars to preserve a failing one. Another, placing a low estimate on the same would be unwilling to pay more than half a dollar to save it. For, at the expense of half a dollar the troublesome member can be got rid of entirely.

Money is demanded as a price, not for any intrinsic value it possesses, but as à mere matter of convenience. What I want is wood, meat, butter, dry goods, house and home. But it is not convenient at a specified time, for me to receive them. So I demanded *money*, which is made the representative of all values, and this enables to obtain the articles needed when and where it is convenient.

There are in the country great staple commodities in which is invested almost the entire capital of the country, and in the exchange and transfer of these the interests of commerce are chiefly engaged. The price of these commodities is regulated on the principle of supply and demand. When supply is less than demand the price goes up—when greater than demand the price goes down. This principle applies to and regulates all articles of prime necessity. In the production of these physical labor and mechanical force are the chief agents.

But outside of and beyond the lists of commercial commodities is a vast range where enlightened, cultivated, and refined society makes its demand and offers its rewards. In the domains of literature, science, art, and the higher grades of artisan skill, there is not the same relation of price to things demanding price. Here price is not regulated by *supply and demand.* Over this vast domain *supply* can never equal *demand.* For the thing demanded is not measured nor weighed, so much for so much. To illustrate: in a field of corn it is days of labor that are sold: in the field of science it is fact and principle that demand the price. The price of manual labor is so much per day: the price of a book is the same whether in the production of it thirty days or one hundred days were required. Because, it is the matter contained in the book that is sold, and not the labor bestowed upon it. In the field of science it is results that command the price. In one case years are spent, a fortune and a lifetime are exhausted without arriving at the desired result. For this society pays nothing. Another by half an hour's thought obtains it. This success is a fortune to him Who will attempt to measure out to him his success as a commodity, and propose pay according to time spent?

The demand of society for scientific research is unlimited. Hence supply can never equal the demand. The demand of society for medical skill is so great that the medical man will never meet it. The demand upon the lawyer and statesman is such that none have ever been wise or learned enough to say, 'we can supply the demand.' For, be it remembered, the demand is not for books, but for instruction—not for printed sheets, but for thoughts—not for lawyers, but for legal intelligence—not for medical men, but for medical ability—not for Dentists, but for dental skill. Let society in any town be questioned as to the demand for more Lawyers, or Doctors, or Dentists, and the answer will be, 'we are supplied.' If the question be as to the demand for more legal intelligence, more medical ability, more dental skill, the reply will be, 'we greatly need it.' Such accessions to the profession in any community would be appreciated and rewarded. A surgeon of wide reputation, based upon a wonderful success in his operations, was invited by a lady to come to her house to open an abscess. The operation was performed in a moment's time and with a surprising delicacy of touch. When the charge of five dollars was announced the lady opened big eyes and wondered how he could charge so much for what had given him so little trouble. He replied "I charge you, madame, not *for what I do*, but *for what I know.*" This, then, is the peg on which the professional fee bill hangs. This surgeon had taught his knife when, where, and how far to cut—to draw blood or pus only, at will. *For this* he charged, whether the knife was employed to open a boil, or to amputate a leg. If a lawyer could do up his opinions in packages, at his leisure, and in advance of the application for them, he could sell his opinions to his clients by the bundle, as a commodity. If a physician could foresee all the sickness which would befall the families of his patrons for years to come, and give his advice and write his prescriptions in advance, he could sell his advice as a commodity. But every case is a special case, demanding attention according to a variety of ever changing circumstances and conditions. So the professional man does not sell his books to his clients and patrons, but *his opinions and advice.* He does not charge for the ten minutes time employed; but for the *advice* itself. So the Dentist does not make his charge on the basis of ten, twenty or fifty pounds of strength employed in a given operation—nor on the scale of a few minutes or a few hours time occupied—nor for high or low priced instruments used—nor chiefly for more or less material consumed—but mainly, for his knowledge of what ought to be done, and for the acquired skill and experience employed in accomplishing it. In medicine this principle is very generally recognized, and a fee is charged whether medicine is given or not. We must by some measures arrive, ultimately, at the same principle in Dentistry—so that when a Dentist confers a greater favor by counsel than by a manual operation he shall be rewarded for it. Where application is made for the extraction of a tooth, and the Dentist advises treatment and gives definite directions for the preservation of the tooth, he certainly is entitled to the same fee for saving the tooth that he would be for sacrificing it. We have arrived, then, in the consideration of the subject, negatively, to these conclusions: that professional fees can not be regulated on the scale of equivalents—nor on the scales of commodities, the price of which change from year to year, from week to week, or even from day to day according to the principle of *supply and demand*; that time and manual labor, while they may modify the rate of professional fees, do not furnish a standard by which to govern the scale.

Let us now turn to the affirmative side of the question.

The ability of man to do, springs from two sources—his physical and his mental powers. From these two classes of powers arise two classes of employments—physical aided by the mental, and mental aided by the physical or mechanical. To the former belong the mechanic arts and the trades. To the latter belong the professions. To success in the former, muscular force and manual dexterity are the starting points. To success in the latter, that development of the mental powers which we style education. We may aver with positiveness that a liberal education is the *sine qua non* of success in any of the professions. A man begins a trade with *tools*—he begins a profession with *books.*

Dentistry takes rank among the more noble of the professions. Not as a pursuit wholly mental like the profession of law; but both mental and manual, like the professions of medicine and surgery—and is governed by the same standards.

In the trades there must ever be a higgling and wriggling and wrangling and running up and down of prices: In the professions, honorable and high minded dealing based upon integrity and professional ability, on the one hand, and an appreciation of invaluable services, on the other hand, render it both distateful and dishonorable to bid and underbid, and make sales of professional ability as a commodity. If this is otherwise with the Dentists in any community, it is because the dentists are tradesmen, and hucksters, and not professional gentlemen. Where this is the case, Dentists must come to a higher appreciation of themselves and their calling, and it will be responded to by a higher respect of the community, and an abundant pecuniary reward.

In some parts of Germany professional fees are not a specific and definite charge, but are left as a point of honor with every man to reward professional services according to his ability and his appreciation of their value. There have been cases in this country of men paying to a Dentist, voluntarily, under the promptings of gratitude and a recognition of superior skill, three, four, or even fifty times the fee charged. This shows that enlightened and cultivated society, which is the particular field inviting Dental operations, does to some extent appreciate skill which relieves from suffering, saves from decay, and restores to use any disordered part ot the human body—and that time and manual labor are not always to be taken into account in making up a charge for professional services.

The minor operations of Dentistry are performed for all classes of society. But the more skillful, difficult, and delicate operations are performed chiefly for the more intelligent and refined part of society. In fact Dentistry as a profession was called into being by this class. Teeth have ever been a studied adornment in elegant society—and the Dentist, to fill his place acceptably, must himself rank socially where his profession finds its most important patronage. To be qualified for such a position in society requires of the Dentist that he be thoroughly educated in his profession—that he keep himself posted in all the improvements and new methods of operating, by taking the best Dental Journals—that he be acquainted in the field of general science and current literature whence cultivated and intelligent society gathers themes of conversation. But all this costs money. It requires an income. Every Dentist, therefore, who sets before him a high standard of professional success, or who has already gained it, must of necessity have in view the *entire cost* when he graduates his fee-bill. His annual income must be such as will enable him to stand on the level of cultivated and refined society that gave birth to his profession—and such will enable him to pursue that profession according to the highest standard of cultivated taste and acquired skill. In this way only can he with the best acceptance serve his patrons. He must not only desire the best success, but so love complete and perfect operations, that this very love of artistic skill in Dentistry will stimulate him to do for his patrons at all times, the best possible. Thus while he guards his professional reputation from decline, he protects his patrons from being dismissed from his office with something from the Dentist's hand of inferior quality, which does not meet their high expectation. But this love of complete manipulation and artistic skill needs itself to be stimulated, or soon the fire goes out. Very few will labor assiduously from the mere love of labor—few pursue science for the mere love of science—few persistently strive to excel from the mere love of perfect operations. The world has universally conceded the value of rewards and premiums even in keeping alive the most enthusiastic love of noble pursuits. When a Dentist knows that his efforts will be appreciated, and rewarded pecuniarily according to the completeness of his work, he labors with increasing earnestness, and is daily outstripping himself in his successes. But on the other hand, if he is poorly compensated for his skill, and he suffers himself to be, in common parlance, *beat down to the lowest figure*, one stimulus *to do his best* is gone—and he at length falls into the habit of grading down the quality of his operations to this low priced level—to his own discredit as a reliable Dentist, to the dishonor of the profession, to the damage of his patrons and to the ultimate damage of his own pecuniary interests. He must strive first of all for a reputation based upon superior dental skill. Having gained it, it is his capital in a professional business which will give him a successful practice any where in spite of all dishonorable competition. When the Dentists of this State will properly appreciate themselves, their calling, and their services, and cease to traffic and barter like market hucksters, we shall arrive at the end of a disgraceful war of prices, and find that in the esteem of the community a well regulated moderate fee-bill will be an honor to every Dentist who subscribes to it—and by so doing we shall sound the note of professional harmony throughout the State.

KEOKUK, Iowa, Aug. 10, 1864.

